# The Role of RECK in Hepatobiliary Neoplasia Reveals Its Therapeutic Potential in NASH

**DOI:** 10.3389/fendo.2021.770740

**Published:** 2021-10-20

**Authors:** Ryan J. Dashek, Connor Diaz, Bysani Chandrasekar, R. Scott Rector

**Affiliations:** ^1^ Research Service, Harry S. Truman Memorial Veterans’ Hospital, Columbia, MO, United States; ^2^ Comparative Medicine Program, Department of Veterinary Pathobiology, University of Missouri, Columbia, MO, United States; ^3^ School of Medicine, University of Missouri, Columbia, MO, United States; ^4^ Division of Cardiology, Department of Medicine, University of Missouri, Columbia, MO, United States; ^5^ Dalton Cardiovascular Research Center, University of Missouri, Columbia, MO, United States; ^6^ Department of Medical Pharmacology and Physiology, University of Missouri, Columbia, MO, United States; ^7^ Department of Nutrition and Exercise Physiology, University of Missouri, Columbia, MO, United States; ^8^ Division of Gastroenterology and Hepatology, Department of Medicine, University of Missouri, Columbia, MO, United States

**Keywords:** RECK, non-alcoholic fatty liver disease, non-alcoholic steatohepatitis, extracellular matrix, inflammation, fibrosis, hepatocellular carcinoma

## Abstract

Non-alcoholic fatty liver disease (NAFLD) is a multimorbidity disorder ranging from excess accumulation of fat in the liver (steatosis) to steatohepatitis (NASH) and end-stage cirrhosis, and the development of hepatocellular carcinoma (HCC) in a subset of patients. The defining features of NASH are inflammation and progressive fibrosis. Currently, no pharmaceutical therapies are available for NAFLD, NASH and HCC; therefore, developing novel treatment strategies is desperately needed. Reversion Inducing Cysteine Rich Protein with Kazal motifs (RECK) is a well-known modifier of the extracellular matrix in hepatic remodeling and transition to HCC. More recently, its role in regulating inflammatory and fibrogenic processes has emerged. Here, we summarize the most relevant findings that extend our current understanding of RECK as a regulator of inflammation and fibrosis, and its induction as a potential strategy to blunt the development and progression of NASH and HCC.

## Introduction

The extracellular matrix (ECM) is a complex and dynamic component of multicellular organisms, regulating crucial cellular processes such as proliferation, differentiation, migration, adhesion, and tissue remodeling (1). As such, dysregulation of the ECM has been linked to several pathological conditions, including cancer and fibrosis (1, 2). Therefore, regulators of the ECM play pivotal roles in these conditions and have been explored as potential therapeutic targets in a variety of diseases. One such regulator is Reversion Inducing Cysteine Rich Protein with Kazal Motifs (RECK), a membrane-anchored glycoprotein (3). At the NH_3_-terminal, there are five cysteine repeats followed by two epidermal growth factor (EGF)-like repeats that are hypothesized to be required for proper interaction between RECK and its targets (4, 5). Moreover, at the COOH terminus, there exists three serine protease inhibitor (SPI)-like domains, that play a role in inhibiting target peptides through ‘physical trapping’ or ‘reversible tight binding’ (4). RECK itself is anchored to the cell membrane *via* the GPI-anchor located at the COOH-terminal (4).

RECK primarily regulates the activity of several matrix metalloproteinases (MMPs) that play a role ECM remodeling (6). This regulation of ECM components, combined with the observation that RECK is downregulated in cancers that metastasize, prompted several studies aimed at understanding RECK’s potential as a metastasis-suppressor (5, 7–9). However, it is not known how RECK regulates inflammation and the fibrogenic pathways.

Both inflammation and fibrosis contribute to the progression of nonalcoholic steatohepatitis (NASH), a clinical condition characterized by the excess accumulation of fat in the liver. It affects a subset of patients with NAFLD (non-alcoholic fatty liver disease) and can ultimately lead to liver fibrosis (cirrhosis) and transition to HCC (hepatocellular carcinoma). NAFLD affects approximately a third of the adult population in developed countries, and NASH is expected to become the leading indicator of liver-related mortality within a few years (10). Unfortunately, to date, no specific pharmacological therapies exist for NASH or HCC. Reduced RECK is a characteristic feature of many cancers, including HCC (11), promoting progression and metastasis. Interestingly, reduced RECK may also be a component of NASH as reported by Peng, et al. (12), where the authors reported that RECK expression is downregulated in mice fed a methionine-choline-deficient diet (12). Furthermore, they found that RECK is a novel target of Farsenoid X Receptor (FXR), and that FXR activation induced RECK mRNA and protein expression (12). In fact, FXR agonists have been identified to inhibit NASH by reducing hepatic gluconeogenesis, lipogenesis, and steatosis (13, 14). The use of therapeutic bile acids as FXR agonists appear promising in human clinical trials, but additional studies related to their long-term safety are warranted (15–17). It is plausible that FXR agonists may improve NASH outcomes, in part, through upregulation of hepatic RECK expression, as hepatic RECK knock-down appears to worsen hepatic inflammation and fibrosis in animal models of Western diet-induced NASH (our unpublished data). Here we seek to describe reduced RECK in the context of hepatocellular inflammation, fibrosis, NASH, and HCC.

## RECK and Cancer

The ECM plays a critical role in the development and progression of cancer. Dysregulated ECM remodeling by tumor cells alters cell signaling, angiogenesis, and tissue biomechanics (18). These changes in the tumor microenvironment allow for local tissue invasion as well as distal metastasis of cancer cells (18). Therefore, regulators of the ECM have drawn much interest in the oncological realm, and RECK, a key player in ECM remodeling, is no exception.

Reduced RECK is a characteristic feature of many cancers (19). In fact, interest in regulators of the gelatinases, MMP2 and MMP9, stems from their identification as prognostic indicators in a number of tumors, including liposarcoma (20), breast cancer (21), oral squamous cell carcinoma (22), and ovarian cancer (23). Tumors expressing higher concentrations of these gelatinases are in general linked to poorer prognosis and overall survival (1, 24, 25). Furthermore, RECK is known to complex with MT1-MMP, and inhibit its proteolytic activity at the cell membrane and internalization (26). Increased expression of MT1-MMP in tumors has also been linked to poorer prognosis and reduced overall survival, independent of other gelatinases (27). The underlying theory is that sustained activation of these gelatinases promotes excess ECM degradation allowing for local and distant tumor invasion, as well as allow for angiogenesis. For example, MMP9 is known to promote the release of vascular endothelial growth factor (VEGF), a pro-angiogenic mediator (4, 19, 28). Interestingly, RECK was first identified as a gelatinase inhibitor, reducing ECM breakdown and promoting angiogenesis in several tumor types (29–32), including colorectal, gastric, and HCC. Across all tumor categories, preserving RECK expression was shown to inhibit MMP2 and MMP9 activity, and improve prognosis by decreasing invasion and metastasis (5).

As mentioned above, RECK expression is either downregulated or undetected in various invasive cancers (4). By contrast, tumors that expressed normal or elevated RECK levels show reduced tissue invasion and metastasis (4). The mechanisms underlying RECK downregulation in cancer are hypothesized to be multifactorial and tumor specific; however, the general mechanism appears to involve increased Sp1 binding to the *RECK* promoter, resulting in its reduced transcription (5). It is, however, unclear whether RECK downregulation occurs prior to, concurrently, or following tumor metastasis. Regardless, reduced RECK expression appears to worsen prognosis (7, 9, 33). RECK has also been shown to interact and inhibit other cellular pathways involved in cancer progression and metastasis, such as Notch and EGFR/RAS. Both Notch (34) and EGFR/RAS (22, 23) are implicated in inflammation and fibrosis, which we will explore further below.

## RECK and Liver-Related Tumorigenesis

Within the context of the liver, RECK’s role was assessed in the pathogenesis of HCC and cholangiocarcinoma (CCA). In line with other oncological studies, several groups have found that overall prognosis and survival were significantly improved when tumors – either HCC or CCA – expressed relatively greater amounts of RECK (11, 29, 33, 35). What remains unanswered, however, is how and when RECK expression is altered in these individuals – *i.e*., does RECK suppression precede tumorigenesis, or occurs during progression and metastasis?

Several mechanisms are implicated in RECK downregulation. For example, several single nucleotide polymorphisms (SNPs) are identified in the *RECK* gene within given populations (36–38). These SNPs appeared more frequently in patients diagnosed with HCC *versus* healthy controls (36, 39), with Chung et al. outlining specific SNPs in *RECK* that are relevant to liver cancer in humans. Their group identified two SNPs of interest in the development of HCC; individuals with the *RECK* promoter rs10814325 polymorphism saw increased risk of developing HCC compared to wild-type carriers, while HCC patients carrying the rs11788747 had higher risk of developing distant metastasis than wild-type carriers (36). This leads to the hypothesis that singular changes within the RECK protein structure itself or promoter polymorphisms could have a significant impact on its activity and ultimately on tumor progression. Hypermethylation of its promoter has also been shown to downregulate RECK expression (33). In addition, hypermethylation of *RECK* promoter led to poorer prognosis in individuals with HCC (33).

Several micro-RNAs (miRs) are also shown to target RECK in HCC. For example, miR-135b, upregulated in HCC tissues, not only targets RECK post-transcriptionally (40), but also promotes HCC cell motility and invasiveness *in vitro* (41). Huang, et al. reported increased miR-21 and reduced RECK expression in CCA patients with lymph node metastasis or perineural invasion (42). In that study, silencing miR-21 dramatically decreased CCA cell invasion and metastasis, which was rescued by the forced expression of RECK.

These studies suggest a direct link between reduced RECK expression and invasion and metastasis of liver cancers. However, several questions remain unanswered. For example, what other mechanisms play a role in RECK regulation, and when RECK expression is altered, *i.e*., is this a dynamic process that can change over time or is activity static and only serves as a predisposing factor in these cases? It is also unclear whether RECK expression is altered prior to the formation of HCC in situations of NAFLD or cirrhosis. Of note, Furumoto et al. found that approximately half of individuals with HCC recruited into the study had reduced RECK expression. However, they did not delineate cases based on predisposing factors leading up to HCC, such as which patient had NASH prior to recruitment into the study (11).

It is unknown whether RECK is already downregulated or silenced in NAFLD, causing exacerbation of symptoms, including development and progression towards NASH, cirrhosis, and HCC. Individual heterogeneity in RECK expression due to various genetic and environmental factors may govern the development of each, or even all these processes. Therefore, we further examined the literature to determine whether RECK was found to be involved in pathways and physiological processes leading up to and including progression of NASH and liver fibrosis.

## RECK and Inflammation

RECK regulation of the ECM also modulates inflammation. For example, RECK/MMP-mediated ECM remodeling plays a role not only in tumor cell spread, but also leukocyte infiltration into tissues. RECK-mediated inhibition of MMP2 and MMP9 expression and activity (19, 43, 44) has been shown to regulate inflammation in a variety of tissues and models. In models of experimental autoimmune encephalomyelitis (EAE), CD4+T cell invasion requires local MMP2 and MMP9-mediated parenchymal basement membrane breakdown (45, 46). MMP2 and MMP9 knock-out (KO) mice have reduced inflammatory cell influx into bronchoalveolar lavage fluid in experimental asthma models (47, 48). In models of acute pyelonephritis, it is known that there is a direct correlation between levels of MMP2 and MMP9 in the kidney and the severity of inflammation (49). Nascimento et al. found that MMP9 was involved in the early phases of temporomandibular joint inflammation in a rodent model, while MMP2 was involved in later phases of inflammation of the joint capsule. Additionally, they found that using doxycycline, a non-specific MMP inhibitor, diminished the inflammatory response (50). Furthermore, MMP9 was established as a mediator of inflammation within the intestinal muscularis in rodent models of post-operative ileus; inhibition of MMP9 activity reduced immune cell infiltration into intestinal muscularis, and MMP9-KO mice were protected from the inflammation and dysmotility associated with post-operative ileus (51). Finally, Ries, et al. found that inflammatory cytokines upregulate MMP2 and MMP9 in cultured human mesenchymal stem cells, which in turn allowed for chemotactic migration through reconstituted basement membranes (52), suggesting a complex interplay between inflammatory cytokines, MMP activity, and immune cell infiltration through a basement membrane. Given such, RECK may be a central regulator in controlling leukocyte extravasation into other tissues as well, such as liver in NASH.

Chronic inflammation in obesity has been shown to closely associate with metabolic syndromes, such as NASH. In the context of obesity and inflammation, elevated MMP2 expression and MMP9 activity are found in a mouse model of obesity and positively correlated with inflammatory cytokine expression (53). Even more compelling is that MMP9 has already been shown to be involved in the active recruitment of CD11b+ leukocytes (54) and migration of neutrophils (55) in the post-ischemic liver. Lingwal, et al. examined swine islet cell transplantation into the liver of C57Bl/6 mice *via* the portal vein and found that the transplantation drove an increase in MMP9 activity, which corresponded with massive inflammation in the liver (56). Using MMP9-KO mice, they found hepatic inflammatory infiltrates were significantly lower. More specifically, a positive correlation was observed between hepatic MMP9 expression and activity and CD11b+ leukocyte infiltration. Further, using pharmacological gelatinase inhibitors *in vitro* and *in vivo*, they reported a significant decrease in Kupffer cell migration towards TNF-α or IL-1β expressing loci (56). These results suggest that the gelatinases, MMP2 and MMP9, are critical in the inflammatory processes of the liver, and, through inhibiting the activity of these matrixins, reduction in inflammatory infiltrates could be achieved. As the downregulation of RECK clearly disrupts ECM integrity in the liver through dysregulation of MMP activity – as evidenced by the spread and invasiveness of HCC and CCA when RECK concentrations are lowered, as well as in the Lingwal, et al. study (56) – we could ask two critical questions that need further investigation: (i) would RECK downregulation lends itself to increased invasion of inflammatory cells into the liver in cases of NAFLD and NASH? and (ii) could restoring RECK reduces the amount of inflammation in these patients?

Beyond MMPs, RECK is also a known inhibitor of ADAM17 (A Disintegrin and Metalloproteinase Domain-containing protein 17) (57). Known also as TNFα-Converting Enzyme (TACE), ADAM17 plays a pivotal role in inflammation (58, 59). Of note, TNF-α expression has been shown to be upregulated in NASH (60), and plays a role in the development and progression of NAFLD (61). Therefore, regulating TNF-α release by targeting ADAM17 may be an effective strategy to blunt hepatic inflammation. However, identifying a pharmaceutical inhibitor of this enzyme has remained a challenge. It is therefore plausible that sustaining or inducing RECK has the therapeutic potential to target ADAM17 and overt inflammation in the liver as a result of metabolic dysregulation.

In addition to ADAM17, RECK has also been shown to inhibit ADAM10, though both ADAMs are critical and play a role in the activation of the pro-inflammatory Notch signaling cascade (34, 57). In fact, RECK has been shown to inhibit Notch signaling in neural tissues (34) and during angiogenesis (62). An increases in the Notch signaling pathway has been implicated in several proinflammatory conditions, such as rheumatoid arthritis (63) and uveitis (64). Increased Notch activity, specifically Notch2, is known to regulate monocyte cell fate and inflammation in response to Toll Like Receptor (TLR) signaling (65). Both canonical and non-canonical Notch activity have been found to be increased in response to inflammatory mediators (66), thereby creating a positive feedback loop of Notch➔inflammation➔Notch signaling. In the realm of NAFLD, the number of hepatocytes expressing a major Notch outcome product – Hes Family BHLH Transcription Factor 1 (Hes1) – is significantly elevated in patients with severe NASH (67), suggesting overt activation of this pathway. Since RECK modulates the Notch pathway *via* direct regulation of ADAM17 and ADAM10, strategies that sustain or induce RECK expression have the therapeutic potential in NASH.

In addition to Notch signaling, both ADAM10 and ADAM17 are shown to be crucial in regulating the epidermal growth factor receptor (EGFR) signaling cascade. For example, RECK’s inhibition of the ADAMs could prevent the release of membrane-anchored EGFR ligands, such as amphiregulin, and suppress EGFR activation. Indeed, RECK’s ability to downregulate EGFR activity has already been reported (68, 69). This is of particular interest in the context of NASH, as EGFR has been implicated in hepatocyte and liver regeneration, and HCC development. EGFR signaling is also implicated in mitochondrial dysfunction, apoptosis of hepatocytes and hepatic stellate cells (HSCs), and liver necrosis (70–72). Pharmacological inhibition of EGFR has shown to reduce high-fat diet-induced liver injury in mouse models of NAFLD (73, 74), suggesting that targeting EGFR signaling may prove to have therapeutic potential in human NASH. Sustaining or inducing RECK may be a strategy to modulate EGFR activity and inhibit NASH.

## RECK and Fibrogenesis

Fibrosis results from excess accumulation of ECM components. The downregulation of RECK has been linked to fibrosis in several tissues. In a mouse model of Western diet-induced obesity, RECK protein levels were found to be decreased in the kidney and correlated positively with renal fibrosis (75). We previously reported reduced RECK expression in the fibrotic heart. We also reported reduced RECK expression and increased angiotensin-II-induced fibroblast migration and proliferation, and their reversal by ectopic RECK overexpression (76–78).

As previously reported by us, RECK regulates fibrosis in part by inhibiting activation of MMP2 and MMP9 (76–78). These gelatinases perform a much wider range of functions than the cleavage of ECM components, and can have more of a ‘processing’ than ‘degradation’ role in maintaining the ECM (79). MMPs have been studied extensively in the context of hepatic fibrosis (80–83). During hepatic fibrogenesis, collagen deposition from HSCs is markedly increased, and paradoxically both MMP2 and MMP9 are highly upregulated in these cells (84). For example, MMP2 is an autocrine proliferator and activator of HSCs (85), promoting further ECM deposition. In an animal model of CCA where RECK was found to be decreased, increased MMP2 was associated with periductal fibrosis (29). Importantly, it was suggested that serum MMP2 levels could serve as a diagnostic marker to assess the level of liver fibrosis in patients with NASH (86). Furthermore, a positive correlation was reported between serum MMP2 concentrations and liver function as assessed *via* bilirubin and albumin production, and prothrombin time (87). While both gelatinases are upregulated in the context of fibrogenesis, paradoxically hepatic fibrosis was exacerbated in MMP2-KO mice (88). This suggests not only a complex relationship between gelatinase function and activity in the context of hepatic fibrosis, but also activation of compensatory mechanisms. Therefore, rather than ablating their expression, inhibiting MMP activity sequentially could blunt progression of fibrosis. As such, sustained RECK expression may have the therapeutic potential in NASH by targeting time-dependent or sequential activation of MMPs.

Notch signaling, and RECK’s modulation of this pathway, may further serve to alter fibrogenesis. Activation of HSCs, classically, is mediated through TGF-β signaling (89), promoting Notch activity and fibrosis. Importantly, pharmaceutical Notch inhibitors prevent TGF-β-mediated HSC activation *in vitro* (90) and limit HSC activation and hepatic fibrosis in an animal model of fibrosis (91). In fibroblasts, Hes1 was shown to promote *Col1A1* and *Col1A2* transcription, and type I collagen deposition (92); however, it is unclear whether this holds true in HSCs as well. As has already been outlined above, RECK inhibits the Notch pathway by targeting ADAM10 and ADAM17 activity; whether this is sufficient to alter fibrosis in NASH patients is unknown.

EGFR signaling is also involved in tissue fibrosis. Its increased activity positively correlated with several pulmonary pathologies; individuals affected by the SARS outbreak of 2003 saw extensive lung fibrosis, which was suggested to be induced by a hyperactive host response to EGFR-mediated lung injury (93). More specifically, in a review by Stolarczyke and Scholte examining chronic obstructive pulmonary disease and cystic fibrosis, extensive evidence was found linking hyperactivity of the EGFR/ADAM17 signaling axis to ADAM17-cleavage of amphiregulin, an EGFR ligand (94). In a rodent model of lung injury resulting from chronic allergies, Morimoto et al. found that amphiregulin/EGFR signaling activated eosinophils to an inflammatory state with enhanced production of osteopontin, an important profibrotic protein. Furthermore, they found that amphiregulin was produced by memory Th cells, further contributing to pulmonary fibrosis (95). Chronic kidney disease (CKD) is associated with fibrosis (96); EGFR is activated following renal injury, and studies have suggested its potential inhibition as a treatment for CKD (97).

In the context of NASH, it has been found that treatment of isolated Kupffer cells, the resident liver macrophages, with CXCL6 increases EGFR phosphorylation and TGF-β induction (98). These results were confirmed by the same authors *in vivo* using a carbon tetrachloride (CCl_4_) model of NASH (98). Increases in EGFR phosphorylation was observed in hepatocytes, activated HSCs, and macrophages in fibrotic livers in response to CCl_4_. Furthermore, *Egfr* gene ablation (EGFR-KO) markedly reduced hepatic fibrosis and α-SMA expression in livers in response to CCl_4_ (99). EGF and EGFR are also upregulated in humans with chronic liver injury. However, in rodent models, it was shown that EGF was downregulated in liver fibrosis, but amphiregulin and EGFR were significantly increased (100). Overall, these reports indicate that overactivation of the EGFR signaling pathway may be linked to overt ADAM17 activity and NASH progression. Due to RECK’s inhibition of ADAM17 and consequent downregulation in EGFR signaling, it is plausible that sustaining or inducing RECK has the potential to prevent or even reverse hepatic fibrosis seen in NASH. A more comprehensive analysis of potential signaling pathway is necessary to better understand the protective role of RECK in NAFLD, NASH and HCC.

## Future Directions

RECK plays a central role in modulating ECM components involved in the progression of inflammation and fibrosis. Therefore, examining the activity of RECK in the context of inflammatory and fibrotic conditions, such as NASH, is paramount. Currently, RECK inducers are being explored in the context of cancer treatment (101, 102), thus expansion of this research into the area of liver disease could prove fruitful and should be considered. Furthermore, there is considerable variation in the context of RECK activity within the individual cell types of the liver. For example, would RECK activity in HSCs prevent activation and collagen deposition? Can RECK activity in Kupffer cells prevent TGF-β release and mobilization through MMP inhibition? Does RECK alter hepatic inflammation and fibrosis through other mechanisms? Further, is RECK expression altered in livers of patients with NASH? Does downregulation predispose individuals towards developing cirrhosis and/or HCC? Further investigations will elucidate RECK’s central role and therapeutic potential in NASH and HCC.

## Conclusion

**Figure 1:**
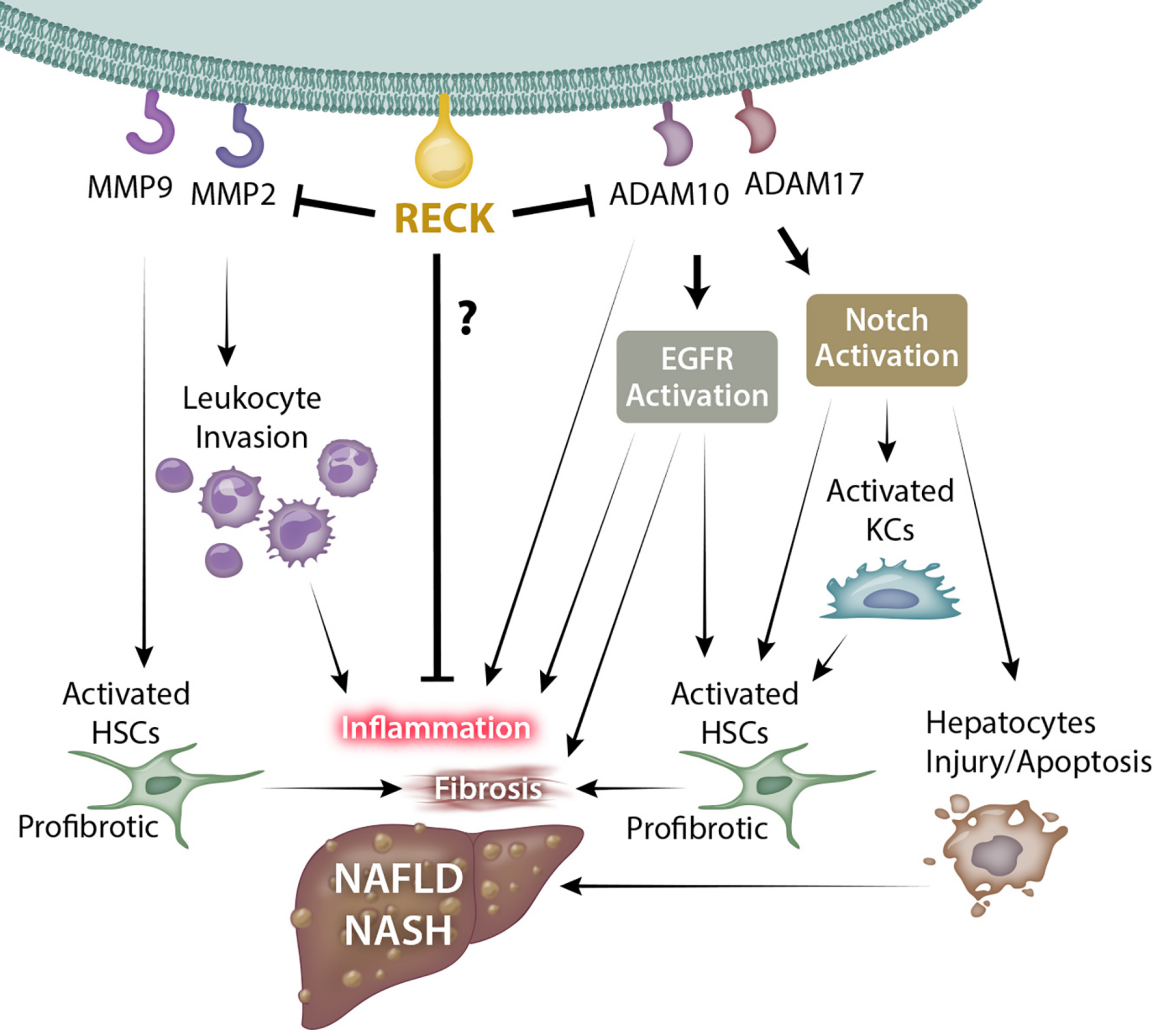
Possible mechanism through which RECK influences NAFLD/NASH development and pathogenesis. RECK’s inhibition of the gelatinases MMP2 and MMP9 may in turn reduce leukocyte invasion into the hepatic parenchyma and hepatic stellate cell activation. In addition, RECK inhibits the sheddases ADAM10 and ADAM17, which consequently may inhibit the release of proinflammatory cytokines from hepatic cells, as well as reduce activation of EGFR and Notch pathways, both of which contribute to inflammation and fibrosis of liver. © Copyright 2021 by The Curators of the University of Missouri, a public corporation.

RECK is a unique membrane anchored regulator of various MMPs and ADAMs. Through modulation of MMPs and ADAMs, RECK could target several key inflammatory and fibrogenic pathways by modulating ECM, inflammatory cytokines, and several other cellular processes, which could influence the outcomes of diseases such as NAFLD and NASH (**Figure 1**; key cellular targets listed in **Table 1**). Further studies are necessary to better understand the regulation and protective role of RECK in the diseased liver. Examination of these pathways may help us develop novel RECK inducers as therapeutics in NAFLD, NASH and HCC.

**Table 1:** Major targets of RECK activity.

Critical Targets of RECK Activity	
Gelatinases (MMP2/MMP9)	Takahashi, 1998 (3), Oh, 2001 (6)
MT1-MMP	Oh, 2001 (6)
ADAM10	Muraguchi, 2007 (28)
ADAM17 (TACE)	Hong, 2014 (51)
Notch Receptor Signaling Pathway	Muraguchi, 2007 (28); Hong, 2014 (51)
EGFR Signaling Pathway	Kitajima, 2011 (62)
Fibroblast migration	Siddesha, 2013 (71); Siddesha, 2014 (72)

## Author Contributions

RD, BC, and RR conceived the manuscript idea. RD and CD wrote the manuscript. BC and RR provided critical editing and input. All authors have read and approved the final manuscript.

## Funding

Support was provided by VA-Merit Grant I01BX003271 and NIH R01 DK113701-01 to RSR, and VA-Merit Grant (I01BX004220) and RCS (IK6004016) to BC. RD is supported by NIH T32 OD011126.

## Conflict of Interest

The authors declare that the research was conducted in the absence of any commercial or financial relationships that could be construed as a potential conflict of interest.

## Publisher’s Note

All claims expressed in this article are solely those of the authors and do not necessarily represent those of their affiliated organizations, or those of the publisher, the editors and the reviewers. Any product that may be evaluated in this article, or claim that may be made by its manufacturer, is not guaranteed or endorsed by the publisher.
